# LRG1 promotes the apoptosis of pulmonary microvascular endothelial cells through KLK10 in chronic obstructive pulmonary disease

**DOI:** 10.18332/tid/186404

**Published:** 2024-05-04

**Authors:** Wei Cheng, Qing Song, Aiyuan Zhou, Ling Lin, Yiyang Zhao, Jiaxi Duan, Zijing Zhou, Yating Peng, Cong Liu, Yuqin Zeng, Ping Chen

**Affiliations:** 1 Department of Pulmonary and Critical Care Medicine, Second Xiangya Hospital, Central South University, Changsha, China; 2 Research Unit of Respiratory Disease, Central South University, Changsha, China; 3 Department of Pulmonary and Critical Care Medicine, Xiangya Hospital, Central South University, Changsha, China; 4 Department of Diagnostic Ultrasound, Xiangya Hospital, Central South University, Changsha, China; 5 Department of Geriatrics, Respiratory Medicine, Xiangya Hospital, Central South University, Changsha, China

**Keywords:** chronic obstructive pulmonary disease, cigarette smoke, pulmonary microvascular endothelial cells, LRG1, KLK10

## Abstract

**INTRODUCTION:**

Cigarette smoking is one of the most important causes of COPD and could induce the apoptosis of pulmonary microvascular endothelial cells (PMVECs). The conditional knockout of LRG1 from endothelial cells reduced emphysema in mice. However, the mechanism of the deletion of LRG1 from endothelial cells rescued by cigarette smoke (CS) induced emphysema remains unclear. This research aimed to demonstrate whether LRG1 promotes the apoptosis of PMVECs through KLK10 in COPD.

**METHODS:**

Nineteen patients were divided into three groups: control non-COPD (n=7), smoker non-COPD (n=7), and COPD (n=5). The emphysema mouse model defined as the CS exposure group was induced by CS exposure plus cigarette smoke extract (CSE) intraperitoneal injection for 28 days. Primary PMVECs were isolated from the mouse by magnetic bead sorting method via CD31-Dynabeads. Apoptosis was detected by western blot and flow cytometry.

**RESULTS:**

LRG1 was increased in lung tissue of COPD patients and CS exposure mice, and CSE-induced PMVECs apoptosis model. KLK10 was over-expressed in lung tissue of COPD patients and CS exposure mice, and CSE-induced PMVECs apoptosis model. LRG1 promoted apoptosis in PMVECs. LRG1 knockdown reversed CSE-induced apoptosis in PMVECs. The mRNA and protein expression of KLK10 were increased after over-expressed LRG1 in PMVECs isolated from mice. Similarly, both the mRNA and protein levels of KLK10 were decreased after LRG1 knockdown in PMVECs. The result of co-immunoprecipitation revealed a protein-protein interaction between LRG1 and KLK10 in PMVECs. KLK10 promoted apoptosis via the down-regulation of Bcl-2/Bax in PMVECs. KLK10 knockdown could reverse CSE-induced apoptosis in PMVECs.

**CONCLUSIONS:**

LRG1 promotes apoptosis via up-regulation of KLK10 in PMVECs isolated from mice. KLK10 promotes apoptosis via the down-regulation of Bcl-2/Bax in PMVECs. There was a direct protein-protein interaction between LRG1 and KLK10 in PMVECs. Our novel findings provide insights into the understanding of LRG1/KLK10 function as a potential molecule in COPD.

## INTRODUCTION

Chronic obstructive pulmonary disease (COPD) is a chronic respiratory disease resulting in respiratory symptoms and airway obstruction that seriously affects human health^1^. But the pathogenesis of COPD is still unclear, and there is no ideal treatment for this disease. Cigarette smoking continues to be identified as the most important risk factor for COPD, the pathogenesis involved with apoptosis, chronic inflammation, oxidation and antioxidation imbalance, and so on^2^. Furthermore, cigarette smoke (CS) exposure causes cell apoptosis, airway inflammation, and pulmonary oxidative stress, which may lead to emphysema and excessive airway mucus secretion^1^. As previously reported, the concentration of nicotine and cotinine was more than 100 ng/mL in the plasma of mice after two weeks of CS exposure^3^. Besides, Krista et al.^4^ revealed that nicotine exposure significantly increased endothelial apoptosis. Previously, we have found that the apoptosis of pulmonary microvascular endothelial cells (PMVECs) was increased in COPD patients and emphysema animal models exposed to cigarette smoke extract (CSE)^5^. Inhibition of the apoptosis of PMVECs can reduce or partially reverse the severity of emphysema in CS or CSE-induced emphysema animal models^5-7^. The above indicated that the abnormal apoptotic events in PMVECs are involved in the occurrence and development of COPD^8^.

The leucine-rich α-2 glycoprotein 1 (LRG1) was first isolated from human serum in 1977^9^. In 1985, researchers determined the amino acid sequence of this protein, with a molecular size of about 50 kDa^9^. LRG1 is a highly conserved member of the protein family rich in leucine repeats (LRR), involved in many protein-protein interactions, signal transmission, and cell adhesion^10^. Previous studies have found that LRG1 was associated with cancer, inflammatory diseases, asthma, neurodegenerative diseases, and emphysema^11-13^. Shu et al.^13^ found that LRG1 was significantly upregulated in pulmonary vascular endothelial cells of human tissues and elastase-induced emphysema mouse model, and the conditional knockout of LRG1 reduced emphysema in model mice. However, the mechanism of the deletion of LRG1 from endothelial cells rescued by COPD or emphysema remains unclear. Previous studies presented that overexpression of LRG1 promotes the apoptosis of cerebral ischemia/reperfusion injury, Lewis lung carcinoma cell lines, and cardiomyocytes in myocardial infarction model^12,14^.

The protein-protein interaction between LRG1 and kallikrein 10 (KLK10) was found by searching the database of protein-protein interaction (https://thebiogrid.org/125537/summary/homo-sapiens/lrg1.html). This phenomenon came from the BioPlex network, which found the protein-protein interaction likelihood between LRG1 and KLK10 in HCT116 cells (human colorectal adenocarcinoma cells) was 99.97%^15^.

The normal epithelial cell-specific-1 (NES 1) gene was first identified in both normal and immortalized mammary epithelial cell lines in 1996. It is a novel serine protease with high homology to the gland kallikrein family seven, and its gene locus is also one for most kallikrein proteins. Based on these, the NES1 gene was named kallikrein-related-peptidase ten, and its encoded protein, KLK10, was a secreted serine protease distributed in the cytoplasm^16^. The expression level of KLK10 in different diseases was also different. The expression of KLK10 was down-regulated during breast cancer progression and prostate cancer tissue^16,17^. However, the expression was upregulated in pancreatic cancer tissues and colorectal cancer cells^18,19^. KLK10 is a flow-sensitive endothelial protein, which is down-regulated in arterial endothelial cells with disordered blood flow^20^. In the past, transcriptome sequencing of lung tissue from Korean male smokers exhibited that the expression level of KLK10 mRNA in lung tissue was correlated to lung function. However, the expression level and function of KLK10 in COPD are still unclear^21^. Current studies have shown that the differential expression of KLK10 could regulate apoptosis in colorectal cancer cells, human prostate cancer cells, and esophageal cancer cells^17,19^.

Therefore, we propose the hypothesis that LRG1 may reduce the apoptosis of PMVECs through KLK10. This research aimed to demonstrate whether LRG1 could promote apoptosis in PMVECs through KLK10 in COPD.

## METHODS

### Human lung tissue

Lung tissue was obtained from patients who underwent pneumonectomy at the Second Xiangya Hospital, Central South University. The exclusion criteria were patients diagnosed with other chronic lung diseases and cancer. Patients were divided into three groups: control non-COPD (n=7), smoker non-COPD (n=7), and COPD (stable stage, n=5). COPD was diagnosed when a ratio of forced expiratory volume in 1 s to forced vital capacity <0.70 after inhaling a bronchodilator^1^. The study was conducted in accordance with the Declaration of Helsinki and approved by the Ethics Committee of the Second Xiangya Hospital of Central South University. Informed consent was obtained from each participant.

### Mice

Six-week-old, male, specific pathogen-free, C57BL/6J mice were purchased from Slyke Jingda Lab Animals corporation (Hunan, China). The animal feeding and experimental protocols were approved by the Experimental Animal Welfare Ethics Committee of Central South University (No. 2021sydw0072). Twelve C57BL/6J mice were randomly divided into two groups: the control group and the CS exposure group (CS exposure plus CSE intraperitoneal injection for 28 days). The exposure method has been reported previously^22^.

### CS exposure plus CSE-induced mouse model

Five unfiltered cigarettes (Baisha, Changde Cigarette Company, Hunan, China) were burned and dissolved in 10 mL of PBS by connecting to a vacuum pump with a constant pressure of -0.1 Kpa. The average burning time per cigarette is one minute. Secondly, CSE was filtered through a 0.22 μm pore-size filter. Finally, CSE was diluted to the concentration of 40% for intraperitoneal injection into mice. The injection details were established as reported previously^23^. The mice in the control group were given 0.3 mL/20 g PBS through intraperitoneal injection, and the mice in the CS exposure group were given 0.3 mL/20 g, 40% CSE through intraperitoneal injection on days 1, 12, and 23. Mice were exposed to total body CS or room air conditions for four weeks. Mice in the control group were treated with room air exposure. CS exposure burning cigarettes (Baisha, Changde Cigarette Company, Hunan, China) was performed using the previously reported CS exposure equipment^7,24^. The mice in the CS exposure group were exposed to CS for two cycles per day, 50 minutes, and 12 cigarettes per cycle, five days per week for 4 weeks^25^. All mice were sacrificed by 1% sodium pentobarbital on day 29.

### Morphometric analysis of lung sections

Lung tissue from humans or mice was fixed in 4% formaldehyde for 24 h at normal temperature, embedded in paraffin to fix, and then sectioned into 3.5 μm thick sections and stained with hematoxylin and eosin (HE). The mean linear intercept (MLI) and alveolar destructive index (DI) were measured as previously described (×100 magnification)^7^.

### Immunohistochemistry (IHC)

The lung tissue of humans or mice was fixed with 4% paraformaldehyde overnight at room temperature, embedded in paraffin, and cut into 3.5 μm sections. 3% bovine serum albumin (BSA) was used to fix lung sections for 15 min after citrate antigen repair buffer (PH6.0) for 15 min in a microwave. Then, sections were incubated with KLK10 (bs-2531R, Bioss, China, 1:100), NF-KB (10745-1-AP, protein tech, USA, 1:200) at 4^o^C overnight. Lung sections were incubated with HRP labeling goat anti-mouse IgG antibody (Servicebio, GB23301, China, 1:200) or HRP labeling goat anti-rabbit IgG antibody (Servicebio, GB23204, China, 1:200) for 50 min at room temperature. Finally, diaminobenzidine (DAB) was added, and hematoxylin was applied for counterstaining. The expression levels were classified into the following grades according to the staining intensity: no staining scored as 0, weak staining scored as 1, moderate staining scored as 2, or strong staining scored as 3. Scored as 0 (0%), 1 (1–10%), 2 (11–50%), and 3 (51–100%) of the staining areas were according to the method of Chiappara et al.^26^ with some modification. The staining index was measured as the product of the proportion of positive cells and the staining intensity score. Comprehensive score 1–3 is low protein expression, and 4–9 is high protein expression. Three different representative non-overlapping fields were selected per lung section.

### TUNEL analysis and immunofluorescence

Apoptosis of mouse lung tissue was identified using the terminal deoxynucleotidyl transferase-mediated dUTP nick end labeling (TUNEL) detection kit (G1501, Servicebio, China) and following the manufacturer’s protocol.

The lung tissue of humans or mice was fixed with 4% paraformaldehyde overnight at room temperature, embedded in paraffin, and cut into 3.5 μm sections. Then, sections were incubated with LRG1 (SC-517443, Santa Cruz, USA, 1:50; SC-390920, Santa Cruz, USA, 1:50), KLK10 (bs-2531R, Bioss, China, 1:50), NF-KB (10745-1-AP, proteintech, USA, 1:200), CD31 (ab182981 Abcam, UK, 1:8000) or SPC (GB114059, Servicebio, China, 1:200) at 4^o^C overnight. Lung sections were incubated with HRP labeling goat anti-mouse IgG antibody (Servicebio, GB25301, China, 1:400) or HRP labeling goat anti-rabbit IgG antibody (Servicebio, GB21303, China, 1:300) for 50 min at room temperature. Then, the reaction solution was added to the lung tissue, and 4’,6-diamidino-2’-phenylindole (DAPI) (G1012, Servicebio, China) was used to counterstain the nuclei. After mounting, we observed the result under a fluorescence microscope (Nikon Eclipse C1, Japan).

### Isolation of mouse PMVECs

We isolated primary PMVECs from a normal 6-week-old male C57BL/6J mouse by magnetic bead sorting method via CD31-Dynabeads (Miltenyi, Germany). Six to eight mice were euthanized with 1% sodium pentobarbital for isolating the lung tissue. The lung tissue was separated under sterile conditions, and the marginal lung tissue within 3 mm was taken and cut into small pieces. We added 30 mL of type I collagenase solution (Sigma, USA, 1.2 mg/mL) dissolved in PBS to the centrifuge tube, and transferred all the supernatant and the lung tissues into a 50 mL centrifuge tube. This tube was placed on a shaker for lung tissue digestion at 37 ^o^C for 45 min. Cells were filtered through a sterile 100 or 40 μm cell strainer and then centrifuged at 1000 rpm for 10 min. Next, we resuspended and washed the cell pellet in MACS buffer. CD31-Dynabeads (20 μL/1×10^7^ cells) were added into the cells and stained on ice for 15 min at 4^o^C. The cells were passed through the LS sorting column (Miltenyi, Germany) and then centrifuged to obtain CD31^+^ PMVECs. The PMVECs were cultured in 5% CO_2_ at 37^o^C with endothelial cell medium (ECM) (Sciencell, USA). The primary PMVECs were identified by von Willebrand factor (vWF, SA00006-4, Proteintech, USA), a marker of vascular endothelial cells, in immunofluorescence staining.

### Preparation of CSE in vitro

According to a previously described protocol with some modification, CSE was freshly prepared on the day of each experiment and used within half an hour *in vitro* experiments^24^. In this research, we tested the optical density (OD) values of 100% CSE to partly maintain the stability of CSE concentrations in *in vitro* experiments^27^. Firstly, one cigarette (Marlboro, Philip Morris, USA) was burned, and the smoke passed through 10 mL of phosphate-buffered saline (PBS) by connecting to a vacuum pump with a constant pressure of -0.1 Kpa. Secondly, the CSE was filtered through a 0.22 μm pore-size filter. The acquired CSE suspension was yellowish with OD 0.233 ± 0.013 at 405 nm. Finally, a concentration of 2% CSE was used in the *in vitro* experiment.

### Small-interfering RNA preparation and transfection

Mouse LRG1 and KLK10 SiRNA interference sequences were designed by Guangzhou RIBOBIO Co., LTD. (Guangzhou, China). Cells were transiently transfected with the LRG1 or KLK10 SiRNA using Lipofectamine TM 3000 (Invitrogen, CA, USA) according to the manufacturer’s protocols. Below are the sequences of these two SiRNA: si-m-KLK10_002, GAAACTGAGCAGTCCTGTA, and si-m-LRG1_002, GCCTACAGCACCTGGATAT. Cells were transfected by replacing the medium with 2 mL of Opti-MEM (Gibco, USA) containing the SiRNA or negative control and Lipofectamine TM 3000 and incubated for 6 h at 5% CO_2_ and 37^o^C. Next, cells were cultured with ECM instead of Opti-MEM for 48 h or 72 h.

### Plasmid preparation and transfection

Mouse LRG1 and KLK10 overexpress plasmid was designed by Genechem Co., Ltd. (Shanghai, China). Cells were transiently transfected with the LRG1 or KLK10 plasmid using Lipofectamine TM 3000 (Invitrogen, CA, USA) according to the manufacturer’s protocols.

The primer sequences of LRG1 were as below:

LRG1_sense primer:

5’-ACGGGCCCTCTAGACTCGAGCGCCACCATGGTCTCTTGGCAGCATCAAGGAAGCCTC-3’

and LRG1-anti-sense primer:

5’-AGTCACTTAAGCTTGGTACCGACAGGGACCCCAGCTCTGCCACGTC-3’.

The primers sequences of KLK10 were as below:

KLK10-sense primer:

5’-GTGGATCCGAGCTCGGTACCCGCCACCATGAGGGTCCCGCTTCTTCAC-3’

and KLK10-anti-sense primer:

5’-ATATTTTATTACCGGTTTAATTAATCATTTGAAACGAATGACTCTTCGGATCC-3’.

The recombinant plasmid (LRG1: CMV-MCS-3FLAG-SV40-Puromycin, Xho I/Kpn I digestion) or KLK10: CMV enhancer-MCS-polyA-EF1A-zsGreen-sv40-puromycin, KpnI/PacI digestion) was transfected into PMVEC cells by lipofectamine 3000TM (Invitrogen, Carlsbad, CA, USA). Cells were transfected by replacing the medium with 2 mL of Opti-MEM (Gibco, USA) containing the plasmid or empty vector and Lipofectamine TM 3000 and incubated for 6 h at 5% CO_2_ and 37^o^C. Next, cells were cultured with ECM instead of Opti-MEM for 48 h or 72 h.

### Real-time quantitative PCR (RT-qPCR)

Total RNA was extracted from human lung tissue or mouse PMVECs using TRIzol reagent (Invitrogen, Carlsbad, CA, USA) according to the manufacturer’s instructions^28^. Total RNA was reverse transcribed using Evo M-MLV RT Kit with gDNA Clean for qPCR II (AG11711, AG, Hunan, China). The qPCR was performed with SYBR green master mix (11184ES08, Yeasen, China) using a StepOne Plus RT-PCR system (Life Technologies, Carlsbad, CA, USA). The primers bellow were synthesized from Sangon Biotech (Shanghai, China):

GAPDH-F (mouse): 5’-CATCACTGCCACCCAGAAGACTG-3’

GAPDH-R (mouse): 5’-ATGCCAGTGAGCTTCCCGTTCAG-3’

LRG1-F (mouse): 5’-CCAATAACTCTCTGTCCAGCACG-3’

LRG1-R (mouse): 5’-TCTTGTTTCGGTTGGCGACCAG-3’

KLK10-F (mouse): 5’-AGGGCAAGAGTGTCAGGTCTCA-3’

KLK10-R (mouse): 5’-AGGTCTCACACTGCTTCTGGCT-3’

GAPDH-F (Homo sapiens): 5’-GTCTCCTCTGACTTCAACAGCG-3’

GAPDH-R (Homo sapiens): 5’-ACCACCCTGTTGCTGTAGCCAA-3’

KLK10-F (Homo sapiens): 5’-GAGTGTGAGGTCTTCTACCCTG-3’

KLK10-R (Homo sapiens): 5’-ATGCCTTGGAGGGTCTCGTCAC-3’.

All samples were assayed in triplicate, and the values were normalized to GAPDH. The quantitative mRNA was normalized by using the 2^-ΔΔCt^ method^29^.

### Western blotting

Total proteins were extracted from the tissues or cells using RIPA buffer (Beyotime, China). The protein concentration of each sample was determined by using the BCA Protein Assay Kit (Thermo Scientific, USA). Protein samples were separated by 10% SDS PAGE. Gels were transferred to 0.45 μm PVDF membrane (Millipore, USA) and incubated with primary antibodies to LRG1 (ab231188, Abcam, UK, 1:3000; SC-517443, Santa Cruz, USA, 1:500), KLK10 (bs-2531R, Bioss, China, 1:500), Bcl-2 (sc-7382, Santa Cruz, USA, 1:500), Bax (14796, Cell Signaling Technology, USA, 1:1000), Cleaved caspase-3 (#9661, Cell Signaling Technology, USA, 1:1000), GAPDH (10494-1-AP, Proteintech, USA, 1:5000), α-Tubulin (66031-1-Ig, Proteintech, USA, 1:10000) at 4°C overnight. The second antibody, HRP-conjugated goat anti-rabbit IgG (SA00001-2, Proteintech, USA, 1:10000) or HRP-conjugated goat anti-mouse IgG (SA00001-1, Proteintech, USA, 1:10000), were incubated with the membranes for 1 h at room temperature. Finally, the bands were visualized with the ECL detection system (ECL; Santa Cruz, CA, USA). The data were normalized to the levels of GAPDH or α-Tubulin.

### Co-immunoprecipitation (Co-IP) and immunoblotting

Total proteins were extracted from the PMVECs using IP buffer, including 0.5M EDTA PH 8.0 (Sangon Biotech, China), NP-40 lysis buffer (Beyotime, China), 1M Tris-HCL PH 7.5 (Beyotime, China), Nacl (Sigma, Germany), and 500 mm DTT (Beyotime, China) for 2 h at 4^o^C. We took 200 μg of the whole-protein lysate as the input and heated it at 100^o^C for 10 min to denature the protein. Then, we separated the remaining total protein lysate into four tubes and added 10 μL Dynabeads^®^ Protein A magnetic beads (10004D, Thermo Scientific, USA) respectively to pre-clearance for 2 h shaking with low speed at 4^o^C. Next, Rabbit mAB IgG (Magnetic bead conjugate) (#8726, Cell Signaling Technology, USA), and KLK10 (PAA697Mu01, Clone, Hubei, China), LRG1 (PAB934Mu02, Clone, Hubei, China) antibodies were added to the supernatant and transferred mixture for 16–24 h shaking with low speed at 4^o^C. A day later, magnetic beads (20 μL per tube) were added to the upper mixtures of KLK10 or LRG1 and shaken at 4^o^C for 2 h. The beads were resuspended with equal proportions of IP and Loading to obtain the IP mixture. Finally, we heated the IP mixture at 100^o^C for 10 min to denature the protein and used the magnetic frame to absorb the magnetic beads and leave the supernatant for immunoblotting.

### Detection of apoptosis by flow cytometry

Annexin V and propidium iodide (PI) staining (Thermo Scientific, USA) were used to analyze cell apoptosis according to the manufacturer’s protocol. PMVECs were resuspended with binding buffer and incubated with Annexin V in the light-avoiding environment for 15 min. Then, cells were incubated with the PI for 10 min. Finally, flow cytometry analysis was performed within 30 min. The apoptosis rates of PMVECs were cells with Annexin V+ and PI-.

### Statistical analysis

Statistical analysis was performed using GraphPad Prism 8.0 (GraphPad Software, San Diego, CA). One-way ANOVA, t-test, or Fisher’s exact test was performed for comparisons among two or multiple groups. A p<0.05 was considered statistically significant.

## RESULTS

### LRG1 protein is over-expressed in lung tissue of COPD patients and CS exposure mouse, and PMVECs of CS exposure mouse

In COPD lung tissue, the protein expression of LRG1, as confirmed by Western blot, was much higher than in control non-COPD lung tissue (Supplementary file Figure 1A).

We constructed the CS plus CSE-induced emphysema model named CS exposure mouse.

Supplementary file Figure 2 A-1 described the body weight changes of the mouse during exposure, and statistical analysis showed differences in body weight changes between the control group and the CS exposure group (p=0.03). The HE staining analysis confirmed that the emphysema indexes, as assessed by the MLI and DI, of mice in the CS exposure group were higher than that in the control group (118.20 ± 12.44 μm vs 57.20 ± 2.60 μm, p<0.0001; 70.00 ± 4.17% vs 21.15 ± 1.16%, p<0.0001, respectively) (Supplementary file Figures 2 A-2 and A-3). The comparison of the above emphysema indexes indicates the successful construction of the emphysema model. The protein expression of Bax and cleaved caspase 3 were higher in the CS exposure mouse lung tissue, and the expression of anti-apoptotic protein Bcl-2 was lower than that in the lung tissue of the control mouse (Supplementary file Figure 2B). Furthermore, immunofluorescence staining of lung tissue samples with TUNEL and endothelial cell marker CD31 revealed the increased apoptosis observed in the PMVECs of CS exposure mice than that in control mice (Supplementary file Figure 3).

We compared the protein levels of LRG1 in CS-exposure mouse lung tissue samples with control mouse lung tissue and found substantially higher levels in the CS-exposure group (Supplementary file Figure 1B). The immunofluorescence staining of lung tissue samples with CD31, a marker of endothelial cells, and LRG1 showed the colocalization of LRG1 in the endothelial population. This immunofluorescence staining also exhibited that the expression of LRG1 was significantly increased in the PMVECs of the CS-exposure mice compared to the control mice (Supplementary file Figure 4).

### LRG1 is over-expressed in PMVECs after CSE intervention

In this experiment, we isolated the primary PMVECs from the lung tissues of normal mice. We present the light microscopic images of PMVECs in Supplementary file Figure 5A. We also used immunofluorescence staining to identify the purity of vascular endothelial cell extraction. Supplementary file Figure 5B shows that the vWF, a marker of vascular endothelial cells, was expressed at nearly 100%, demonstrating successful primary PMVECs extraction.

Initial experiments tested the effect of different concentrations of CSE for 24 h on cell apoptosis in PMVECs using flow cytometry analysis (Supplementary file Figure 6A). We observed a dose-dependent cell apoptosis in PMVECs that was obvious at 2% CSE (3% CSE vs 2%, p>0.05). The concentrations of 2% CSE were used in all subsequent experiments. Furthermore, as shown in Supplementary file Figure 6B, the protein expression of Bax and cleaved caspase 3 were higher in PMVECs after CSE intervention, and the expression of anti-apoptotic protein Bcl-2 was lower than that in the control PMVECs.

The LRG1 mRNA and protein expression were increased in PMVECs after CSE intervention (Supplementary file Figures 1C and 1D). However, we did not observe a dose-dependent LRG1 expression in mouse PMVECs.

### LRG1 promotes PMVECs apoptosis in vitro

**Figure 1:**
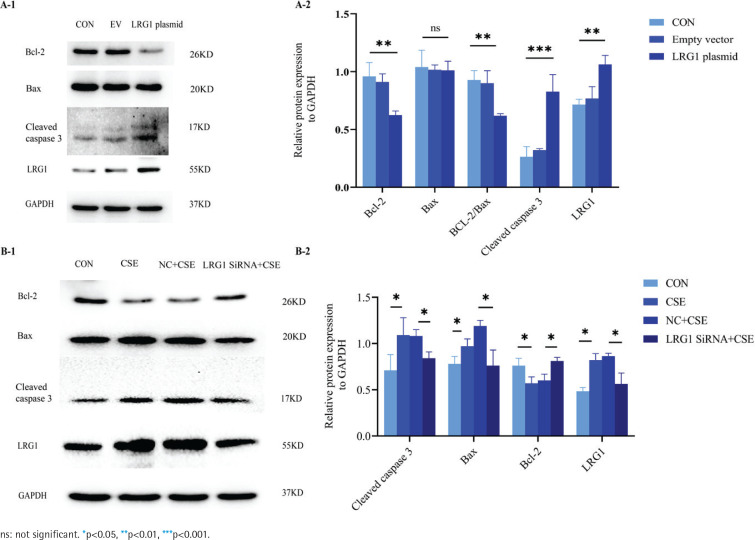
The function of LRG1 in mouse PMVECs: A-1) The protein expression of Bcl-2, Bax, and cleaved caspase 3 between the control group, empty vector group, and LRG1 plasmid group in PMVECs detected by Western blot; A-2) Comparison of the expression of Bcl-2, Bax, Bcl-2/Bax, and cleaved caspase 3 between the control group, empty vector group, and KLK10 plasmid group in PMVECs; B-1, B-2) LRG1 SiRNA reversed CSE-induced apoptosis in mouse PMVECs detected by Western blot

We used the LRG1 overexpression plasmid to mimic the effect of the CSE-induced cell apoptosis in PMVECs. As shown in Figure 1A, the anti-apoptotic proteins Bcl-2 and Bcl-2/Bax ratio decreased more than those in the control and empty vector groups. There was no change in the protein expression of Bax after the transfection of LRG1 plasmid in PMVECs. We found that the apoptosis-related protein cleaved caspase 3, downstream of Bcl-2/Bax, was significantly increased in the overexpressing plasmid group than in the control and empty vector groups. We further detected the apoptosis rate by flow cytometry.

Supplementary file Figure 7 demonstrates that the early apoptosis rate was increased in the LRG1 plasmid group compared to the control group. This indicated that LRG1 may promote apoptosis in PMVECs via Bcl-2/Bax.

### LRG1 knockdown reverses CSE-induced PMVECs apoptosis in vitro

We transfected LRG1 SiRNA for 72 h with PMVECs isolated from normal mice resulting in 60–70% and 30–40% reduction in the respective mRNA and protein (Supplementary file Figures 8A and 8B). The protein expression of Bax and cleaved caspase 3 were lower in the LRG1 SiRNA plus CSE group than in the negative control group plus CSE group. The down-regulation of the anti-apoptosis protein of Bcl-2 by CSE was partly reversed and close to baseline levels (Figure 1B). We further detected the apoptosis rate using flow cytometry. Supplementary file Figure 7 shows that the apoptosis was partially reversed in the LRG1 SiRNA plus CSE group compared to the negative control group plus CSE group. These results indicate that LRG1 SiRNA could partially reverse the apoptosis of PMVECs induced by CSE.

### LRG1 regulates the mRNA and protein expression of KLK10 in PMVECs

**Figure 2:**
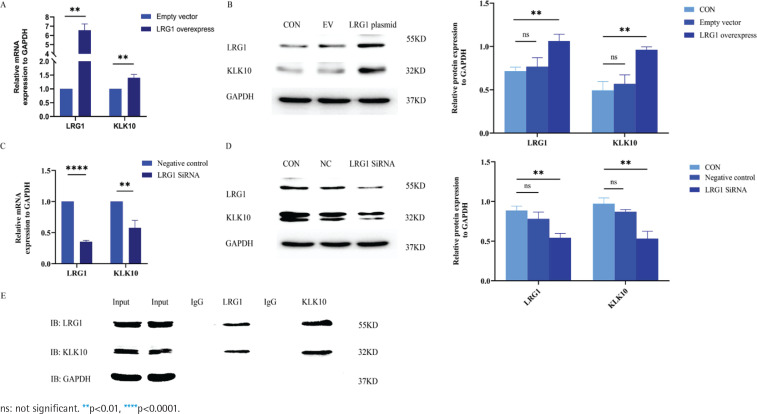
LRG1 regulated the expression levels of KLK10, and the protein-protein interaction between LRG1 and KLK10 in PMVECs: A) The mRNA expression of KLK10 was higher in the LRG1 overexpression plasmid group than in the empty vector group; B) The protein expression of KLK10 was higher in the LRG1 overexpression plasmid group than in the control group and empty vector group; C) The mRNA expression of KLK10 was decreased in the LRG1 SiRNA group than in the negative control group; D) The protein expression of KLK10 was decreased in the LRG1 SiRNA group compared to the control group and negative control group; E) Co-immunoprecipitation of LRG1 and KLK10 in the primary PMVECs isolated from normal mice

To further validate the effects of LRG1 overexpression and silencing on the KLK10 transcript expression levels, we used the LRG1 overexpression plasmid and LRG1 SiRNA for intervening PMVECs, respectively. As presented in Figures 2A and 2B, the mRNA and protein expression of KLK10 were higher after over-expressed LRG1 in the primary PMVECs isolated from normal mice. Similarly, both the mRNA and protein levels of KLK10 were significantly decreased after silencing LRG1 in the mouse PMVECs (Figures 2C and 2D).

### The protein-protein interaction between LRG1 and KLK10 in PMVECs

We further verified the protein-to-protein interaction between LRG1 and KLK10 by co-immunoprecipitation in the primary PMVECs isolated from normal mice. The result of co-immunoprecipitation revealed a direct interaction between LRG1 and KLK10 in mouse PMVECs (Figure 2E). In addition, in Supplementary file Figure 9, we observed colocalization of LRG1 and KLK10 in lung tissues extracted from patients and mice using immunofluorescence dual staining.

### KLK10 is over-expressed in lung tissue and PMVECs of COPD, lung tissue of CS exposure mouse, and PMVECs after CSE intervention

**Figure 3:**
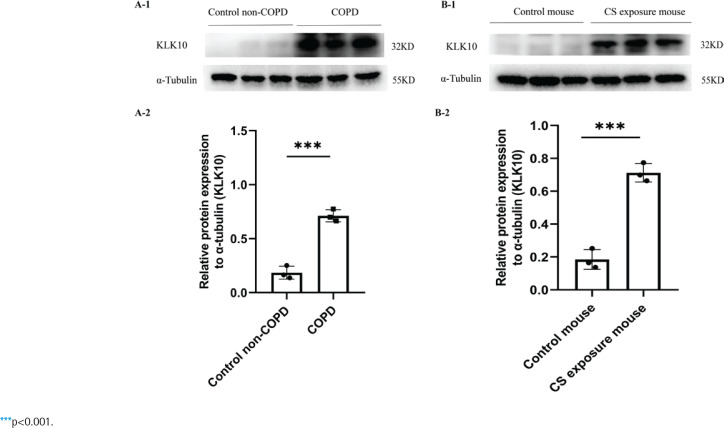
KLK10 is over-expressed in lung tissue of COPD patients and CS exposure mice: A-1, A-2) The expression of KLK10 between lung tissue of control non-COPD and COPD detected by Western blot (n=3 per group); B-1, B-2) The expression of KLK10 between lung tissue of control mouse and CS exposure mouse detected by Western blot (n=3 per group)

The demographic and clinical characteristics of control non-COPD, smoker non-COPD, and COPD patients are presented in Table 1. In COPD and smoker non-COPD lung tissue, the percentage of high protein expression of KLK10 was much higher than in control non-COPD detected by immunohistochemistry (Supplementary file Figure 10A). The mRNA expression of KLK10 was also higher than control non-COPD detecting by qPCR (Supplementary file Figure 10B). Western blotting further clarifies that the protein expression of KLK10 was significantly higher in COPD lung tissue than that in control non-COPD (Figure 3A). The immunofluorescence staining of human lung tissue samples with CD31, a marker of endothelial cells, and KLK10 exhibited the colocalization of KLK10 in the pulmonary endothelial population and the distribution of KLK10 in the cytoplasm (Supplementary file Figure 11A). This immunofluorescence staining also showed that the expression of KLK10 was significantly increased in the PMVECs of human COPD lung tissue than in control non-COPD. However, we did not observe the colocalization of KLK10 in the alveolar epithelial population (Supplementary file Figure 11B).

**Table 1:** The clinical characteristics of patients, 2023 (N=19)

*Characteristics*	*Total (N=19) n (%)*	*Control non-COPD (N=7) n (%)*	*Smoker non-COPD (N=7) n (%)*	*COPD (N=5) n (%)*	*p*
**Sex**					0.124
Female	9 (47.4)	5 (71.4)	1 (14.3)	3 (60.0)	
Male	10 (52.6)	2 (28.6)	6 (85.7)	2 (40.0)	
**Age** (years), mean ± SD	57.3 ± 11.4	56.9 ± 11.2	56.6 ± 15.2	59.0 ± 7.2	0.936
**Smoking status**					**<0.001**
Never smoker	11 (57.9)	7 (100)	0 (0)	4 (80.0)	
Ever smoker	8 (42.1)	0 (0)	7 (100)	1 (20.0)	
FEV_1_/FVC, mean ± SD	74.5 ± 9.6	80.3 ± 3.7	78.4 ± 4.7	61.1 ± 7.0	**<0.001**
FEV_1_, mean ± SD	2.3 ± 0.7	2.7 ± 0.7	2.4 ± 0.6	1.7 ± 0.6	**0.043**
FEV_1_%predicted, mean ± SD	92.0 ± 22.4	112.0 ± 10.6	89.6 ± 18.5	67.2 ± 10.1	**<0.001**

COPD: chronic obstructive pulmonary disease. FEV_1_: forced expiratory volume in 1 s. FVC: forced vital capacity.

In the lung tissue of CS-exposure mice, the protein expression of KLK10 was much higher than that of the control mouse, detected by immunohistochemistry (Supplementary file Figure 10C) and Western blotting (Figure 3B). In PMVECs after CSE intervention, the KLK10 mRNA expression was higher in 3% CSE than in 1% CSE and 2% CSE groups, but there was no difference between 1% CSE and 2% CSE group (Supplementary file Figure 12A). The KLK10 protein expression was increased in mouse PMVECs after CSE intervention (Supplementary file Figure 12B). However, we did not observe a dose-dependent KLK10 protein expression in mouse PMVECs.

### KLK10 promotes PMVECs apoptosis in vitro

**Figure 4:**
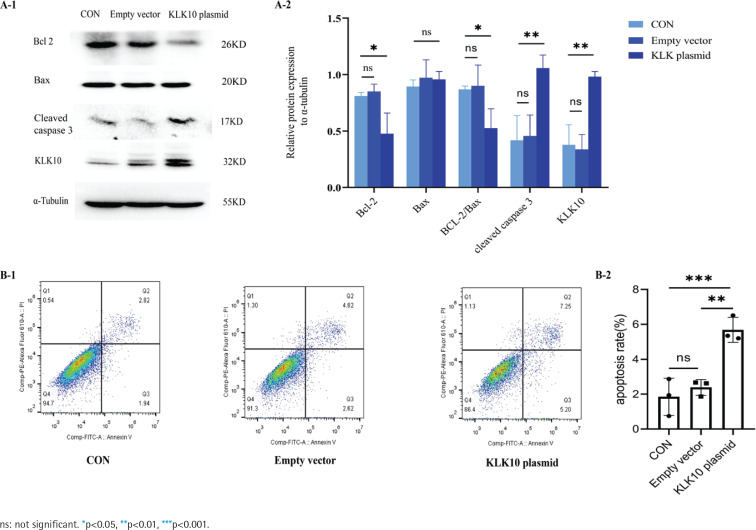
KLK10 promotes mouse PMVECs apoptosis in vitro: A-1) The protein expression of Bcl-2, Bax, and cleaved caspase 3 between the control group, empty vector group, and KLK10 plasmid group in PMVECs detected by Western blot; A-2) Comparison of the expression of Bcl-2, Bax, Bcl-2/Bax, and cleaved caspase 3 between the control group, empty vector group, and KLK10 plasmid group in PMVECs; B-1, B-2) Apoptosis rate in PMVECs between the control group, empty vector group, and KLK10 plasmid group measured by flow cytometry

We used the KLK10 overexpression plasmid to mimic the effect of the CSE-induced cell apoptosis in PMVECs. As exhibited in Figure 4A, the anti-apoptotic proteins Bcl-2 and Bcl-2/Bax ratio decreased more than those in the control group and empty vector group. We found that the apoptosis-related protein cleaved caspase 3, downstream of Bcl-2/Bax, was significantly increased in the overexpressing plasmid group than in the control group and empty vector group. Figure 4B showed that the early apoptosis rate was higher in the KLK10 plasmid group than in the control group and empty vector group. These indicated that KLK10 can promote apoptosis in PMVECs via Bcl-2/BAX (Supplementary file Figure 13).

### KLK10 knockdown reverses CSE-induced PMVECs apoptosis in vitro

**Figure 5:**
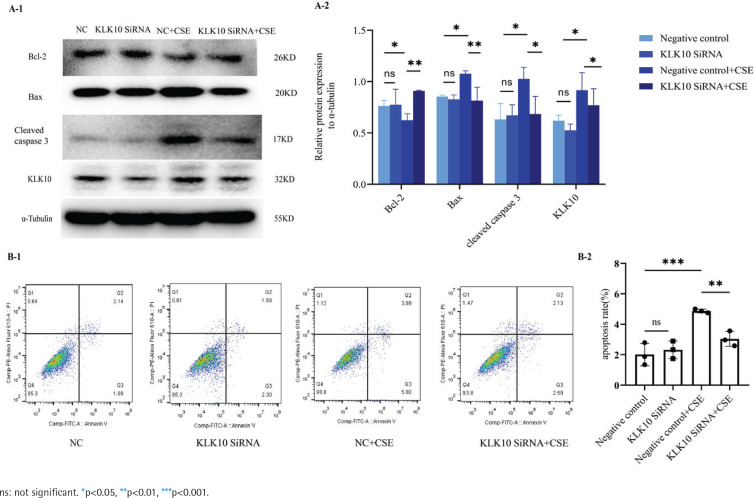
KLK10 knockdown reverses CSE-induced PMVECs apoptosis in vitro: A-1, A-2) KLK10 SiRNA reversed CSE-induced apoptosis in mouse PMVECs detected by Western blot; B-1, B-2) KLK10 SiRNA reversed CSE-induced apoptosis in mouse PMVECs measured by flow cytometry

To test the underlying role of KLK10 knockout in CSE-induced apoptosis of PMVECs, we transfected KLK10 SiRNA for 72 h with PMVECs isolated from normal mice, resulting in 47–56% and 34–42% reduction in the respective mRNA and protein (Supplementary file Figures 12C and 12D). Western blotting clarifies that the protein expression of Bax and cleaved caspase 3 were lower in the KLK10 SiRNA plus CSE group than in the negative control group plus CSE group. The down-regulation of the anti-apoptosis protein of Bcl-2 by CSE was reversed and reached baseline levels (Figure 5A). Figure 5B showed that the apoptosis was partially reversed in the KLK10 SiRNA plus CSE group compared to that in the negative control group plus CSE group detected by flow cytometry. These results indicate that KLK10 SiRNA could partially reverse the apoptosis of PMVECs induced by CSE.

## DISCUSSION

To our knowledge, tobacco smoking is one of the most important causes of COPD, and its pathogenesis involves apoptosis, chronic inflammation, oxidation and antioxidation imbalance, and so on^2^. Furthermore, tobacco smoke exposure could induce the apoptosis of vascular endothelial cells^8^. In this research, we found that LRG1 and KLK10 were upregulated and co-localized in the lung tissue of cigarette-smoking-exposed mice. This study is the first to explore the mechanism of LRG1 in regulating PMVECs apoptosis in COPD. It is also the first time to explore the role of LRG1/KLK10 in the apoptosis of PMVECs in COPD.

Previous studies showed that LRG1 was upregulated in lung tissues and pulmonary endothelial cells of COPD patients. The expression of LRG1 was directly correlated with the percentage of lung emphysema and FEV_1_^13^. However, the colocalization of LRG1 was not observed in alveolar epithelial cells, which suggests that the endothelial LRG 1 population has an important role in the development of COPD. This study also exhibited that the conditional knockdown of endothelial LRG1 alleviated the emphysema severity in elastase-induced emphysema model mouse^13^. There was no consolidated method regarding the CS exposure-induced emphysema model. Our research replicated this emphysema mouse model (CS exposure for four weeks plus intraperitoneal injection of CSE-induced mouse) as previously described^22^. We clarified that LRG1 was upregulated in this CS-exposure mouse lung tissue and pulmonary endothelial population.

Jin et al.^12^ revealed that LRG1 increased apoptosis through the TGFβ-smad1/5 signaling pathway to promote ischemia and reperfusion injury. Takemoto et al.^14^ found a similar apoptosis mechanism of LRG1 in Lewis lung carcinoma mice. In addition, LRG1 could interact with endothelial glycoprotein, ALK, TGFβR1, and TGFβR2 proteins to regulate the angiogenesis of human umbilical vein endothelial cells^10^. However, the mechanism of LRG1 in the apoptosis of endothelial cells is still unclear. We further demonstrated that LRG1 promotes apoptosis in PMVECs via Bcl-2/Bax. And LRG1 SiRNA could partially reverse the apoptosis of PMVECs induced by CSE. In our previous study, inhibition of the apoptosis of PMVECs can reduce or partially reverse the severity of emphysema in animal models^5-7^. Deletion of LRG1 may reduce or partially reverse the severity of emphysema in model mouse by inhibiting the apoptosis of PMVECs.

Next, we aimed to investigate further the mechanism of LRG1 regulating the apoptosis of primary mouse PMVECs. The possible interaction proteins of Homo sapiens LRG1, including KLK10, AGPAT1, NME2, CNPY3, CCR1, CYCS, etc., were obtained from the protein-protein interaction database. To our knowledge, combining cytochrome C (CYCS) and apoptosis activating factor-1 (Apaf-1) can lead to caspase-dependent and independent apoptosis signal transduction^30^. Jemmerson et al.^31^ revealed that intracellular LRG 1 could compete with Apaf-1 for binding to CYCS and protect MCF-7 breast cancer cells from apoptosis. Furthermore, serum LRG 1 competition with Apaf-1 to bind CYCS could alleviate CYCS-induced extracellular lymphocyte death released from apoptotic cells^32^. We have exhibited that LRG1 promotes apoptosis in primary PMVECs in mice. This protective effect of the protein interaction between LRG1 and CYCS in cell apoptosis contradicts our research purpose. We believe there may be a mechanism to protect cell apoptosis, which may be related to the regulation of the cell’s apoptosis balance. By further searching, we found that KLK10, a rare COPD-related protein, had a 99.97% probability of protein-protein interaction with LRG1 in HCT116. We also demonstrated a direct interaction between LRG1 and KLK10 in mouse PMVECs detected by co-immunoprecipitation.

Lee et al.^21^ reported that the KLK10 region of differential methylation was related to FEV_1_ in an epigenome-wide association analysis from the COPD blood cohort. Furthermore, they revealed that the transcriptome sequencing of lung tissue from Korean male smokers (Asan Biobank) showed that the expression level of KLK10 mRNA in lung tissue was correlated to lung function. However, they found no differences in the expression of COPD and non-COPD in KLK10 in the transcriptomic profiles^21^. We think it may be related to the smoking history in both of the non-COPD and COPD male patients, and all of the patients included were mild-to-moderate COPD patients. In our study, we also found no differences between smoking non-COPD and COPD in the expression levels of KLK10 mRNA and protein, which is consistent with their results. A novel finding in our research was that in the lung tissue of COPD, the expression levels of KLK10 mRNA were significantly higher than in non-smokers and non-COPD. If they had used non-smoker non-COPD patients as controls, perhaps the KLK10 mRNA expression could be found to be upregulated in COPD. In addition, the expression level of KLK10 protein expression in COPD is not clear yet. Our results clarified that the protein expression of KLK10 was significantly higher in COPD lung tissue than in control non-COPD. Moreover, the KLK10 protein expression in the PMVECs of COPD was also significantly higher than that in control patients, but no colocalization was observed in the alveolar epithelial population. Previous studies also found that LRG1 was deeply correlated with the pulmonary vascular remodeling pathway. The colocalization of LRG1 was observed in the pulmonary endothelial population, but no colocalization was observed in the alveolar epithelial population^13^. These further suggest that LRG1 and KLK10 are mainly regulated in pulmonary endothelial cells rather than epithelial cells in distal lung tissue cells.

Apart from all the results above, we showed that the protein expression of KLK10 was much higher in the lung tissue of the CS-exposure mouse than in the control mouse. The KLK10 mRNA expression was higher in 3% CSE than in other groups, but there was no difference between the 1% CSE and 2% CSE groups. We have tried to intervene PMVECs with a concentration of CSE of more than 3%, but because the cells cannot tolerate a high concentration of CSE, the majority of cells died, and mRNA detection cannot be completed by qPCR. The KLK10 protein expression was increased in the CSE-induced PMVECs apoptosis model, but we observed no dose-dependent KLK10 protein expression in this experiment. The difference of 3% CSE concentration between KLK10 mRNA and protein levels may be related to the post-transcriptional modification of mRNA. There was no significant difference in KLK10 expression between different concentrations of protein level, so we also chose a 2% concentration of CSE for the subsequent functional test of KLK10 *in vitro*. We have demonstrated that KLK10 SiRNA could partially reverse the apoptosis of PMVECs induced by CSE. In addition, the mRNA and protein expression of KLK10 were higher after over-expressed LRG1 in the primary PMVECs isolated from normal mice. Similarly, both the mRNA and protein levels of KLK10 were significantly decreased after silencing LRG1 in the mouse PMVECs. These suggest that LRG1 could regulate the apoptosis of CSE-induced PMVECs via KLK10. In addition to the effects of the transcriptome and protein levels, these two molecules may also affect endothelial cell apoptosis through protein-protein interactions.

It has been reported that KLK10 may regulate apoptosis in cancer^17,19^. We used the KLK10 overexpression plasmid to mimic the effect of the CSE-induced cell apoptosis in PMVECs. After overexpression of KLK10, we found that the expression of Bcl-2 decreased but Bax did not change. These results indicated that KLK10 mediated the apoptosis through Bcl-2/Bax and then activated the downstream pathway of apoptosis, leading to an increased expression of the apoptotic protein cleaved caspase 3^33^. Previously it has been reported that KLK10 promotes apoptosis of PC3 cells (one of the prostate cancer cells z via the down-regulation of Bcl-2). However, the cleaved caspase-3 protein was not detected in KLK10-expressed PC3 cells. They clarified that KLK10 may inhibit PC3 cell proliferation via caspase-independent cell apoptotic pathway^17^. As previously reported, the Bcl-2/Bax plays an important role in regulating caspase-dependent and caspase-independent apoptosis mediated by the mitochondrial pathway^34^. Importantly, after the knockdown of KLK10 in PMVECs, the down-regulation of Bcl-2 anti-apoptotic protein by CSE was reversed and reached the baseline level, and the expression level of Bax was also decreased, which demonstrated that KLK10 promoted apoptosis via Bcl-2/Bax in PMVECs.

KLK10 also plays a role in inhibiting inflammation, and it is worth noting that COPD can also be mediated by chronic inflammation^20,23^. NF-kB is one of the indicators representing the pulmonary inflammation of COPD^35^. We demonstrated that the expression of NF-kB in the lung tissue of COPD patients was significantly higher than that in control non-COPD (Supplementary file Figure 14A). The NF-kB expression in the lung tissues of the CS-exposure mouse was significantly higher than that of the control mouse (Supplementary file Figure 14B). The colocalization of NF-kB was observed in the pulmonary endothelial population via immunofluorescence dual staining in lung tissues extracted from both patients and mice in Supplementary file Figures 15A and 15B. According to the result of Figure 2E, a protein-protein interaction was observed between LRG1 and KLK10 in endothelial cells. Furthermore, we presented the simultaneous presence of these two proteins via immunofluorescence dual staining in lung tissues extracted from both patients and mice (Supplementary file Figures 9A and 9B). The above indicate that there may be an interrelationship among LRG1, KLK10, and pulmonary inflammation in COPD and endothelial cells.

### Limitations

Our study has some limitations. Firstly, due to the limited number of smoking COPD patients, we could not compare the difference in KLK10 mRNA expression levels between smoking COPD and smoking non-COPD patients. The correlation analysis of KLK10 mRNA expression level and lung function parameters could not be performed in COPD patients due to a similar limitation. Secondly, the mechanism of KLK10 mediating the apoptosis of PMVECs in COPD needs to be further explored in the future. Lastly, further studies are needed on whether the interaction between LRG1 and KLK10 affects the mutual binding of other proteins to the above two proteins and thus affects cell apoptosis. The exact mechanism of this direction will be explored in further experiments.

## CONCLUSIONS

LRG1 and KLK10 were increased in PMVECs of COPD patients, lung tissue of CS exposure mouse, and CSE-induced PMVECs apoptosis model. LRG1 promotes apoptosis via up-regulation of KLK10 in primary PMVECs isolated from normal mice. There was a direct protein-protein interaction between LRG1 and KLK10 in mouse PMVECs. KLK10 promotes apoptosis in mouse PMVECs via the down-regulation of Bcl-2/Bax. Therefore, our study provides insights into the understanding of LRG1/KLK10 function as a potential molecule in COPD.

## Supplementary Material



## Data Availability

The data supporting this research are available from the authors on reasonable request.
