# Supplementary material for: LRG1 promotes the apoptosis of pulmonary microvascular endothelial cells through KLK10 in chronic obstructive pulmonary disease
Source: Tob Induc Dis. 2024 May 4;22:10.18332/tid/186404. doi: 10.18332/tid/186404 (PMC11069109; doi:10.18332/tid/186404)
Supplement: Supplementary file 1 [file TID-22-72-s1.pdf]

Supplementary Figure 1. The expression of LRG1 in *lung tissue of COPD patients and CS exposure mouse, and* mouse PMVECs treated with CSE, 2023

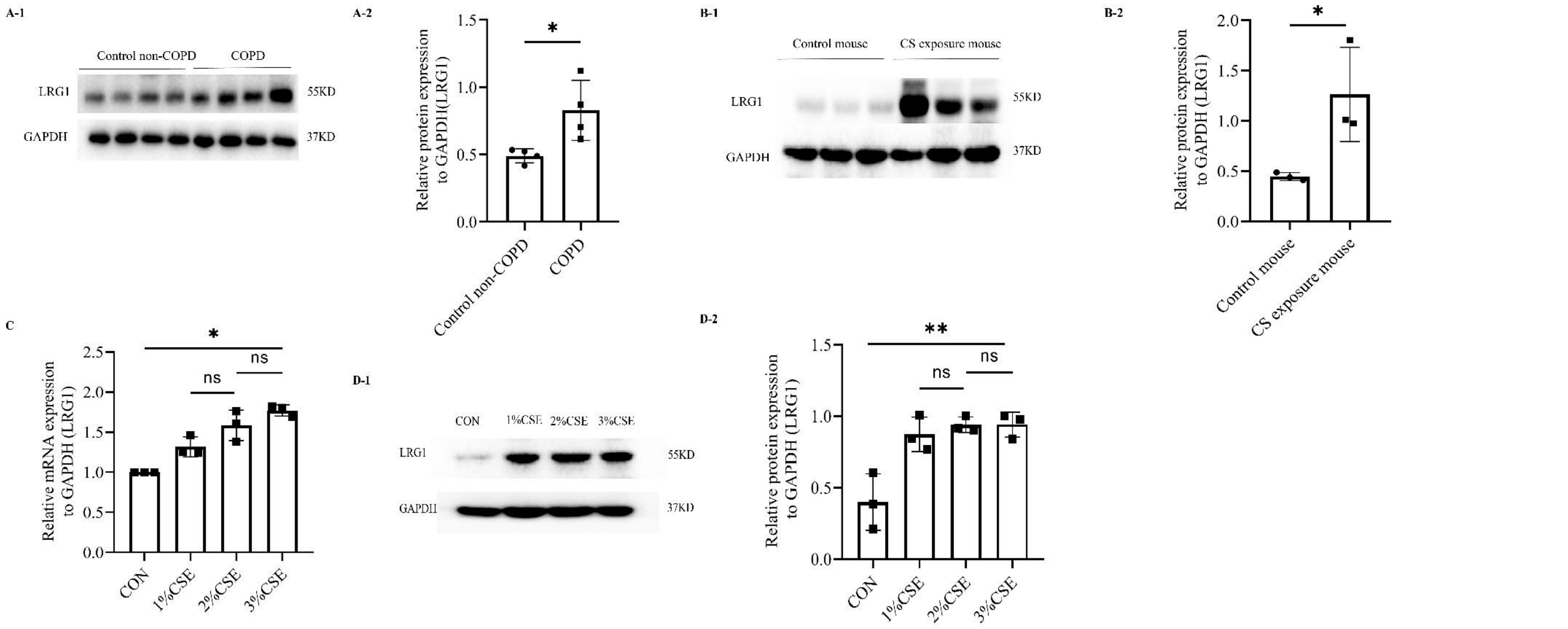

Abbreviations: A. (A-1, A-2) The expression of LRG1 between lung tissue of control non-COPD and COPD patients detected by Western blot (n=4, per group). B. (B-1, B-2) The expression of LRG1 between lung tissue of CS exposure mouse and control mouse was detected by Western blot (n=3, per group). C. The LRG1 mRNA expression in PMVECs treated with different concentrations of CSE. D. (D-1, D-2) The LRG1 protein expression in PMVECs after treated with different concentrations of CSE. ns, not significant, *, $P < 0.05$, **, $P < 0.01$.

Supplementary Figure 2. Construction of the animal model with lung emphysema and apoptosis in PMVECs, 2023

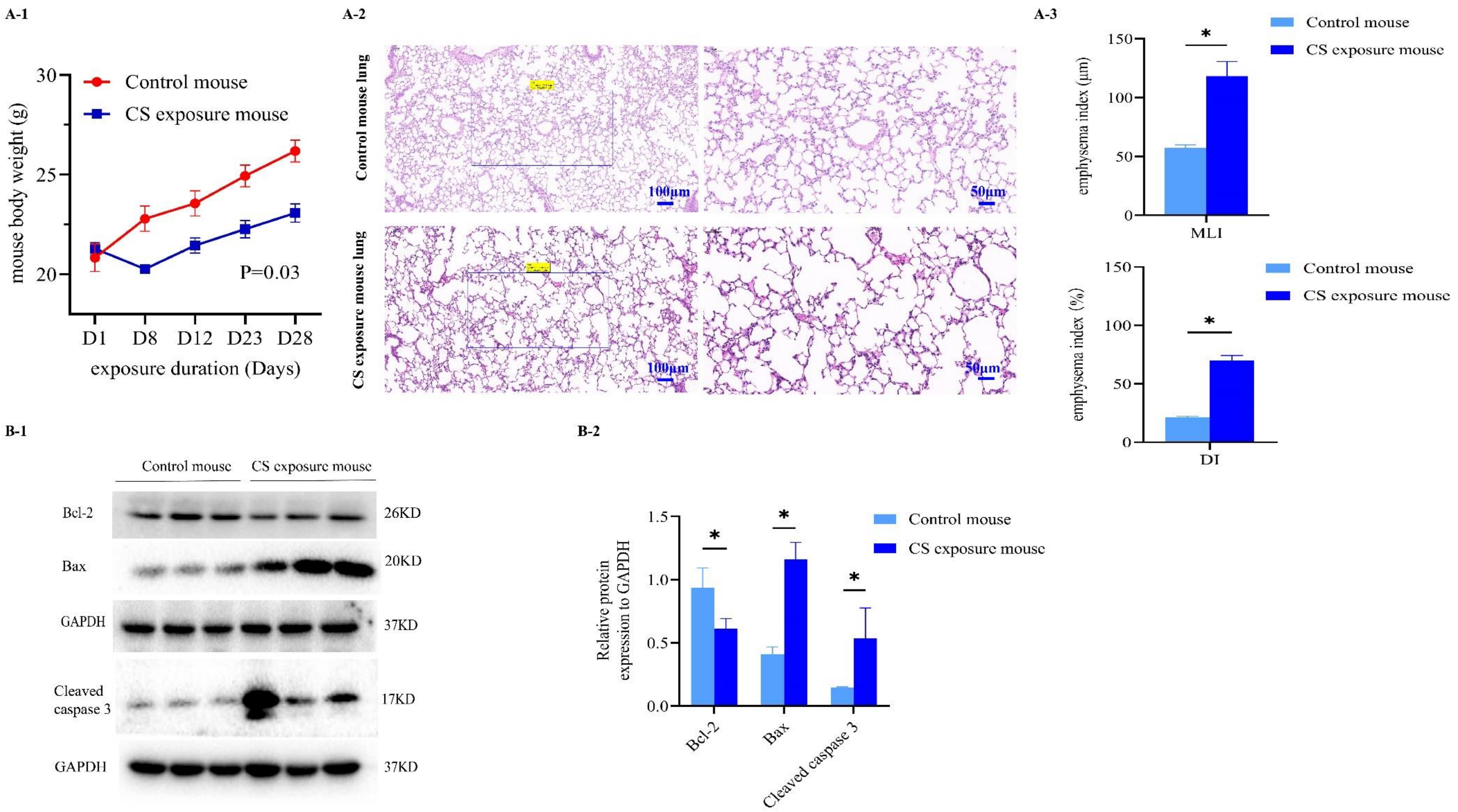

Abbreviations: A. (A-1) The body weight changes of the mouse in the control mouse group and CS exposure group during exposure. (A-2) The hematoxylin and eosin staining of lung sections 28d after CS and CSE instillation (CS exposure group) and 28d after room air and PBS instillation (control group, n=3, per group). scale bar, 100μm (left panel, ×100); scale bar, 50μm (right panel, ×200). (A-3) Statistical analysis of MLI and DI and between lung sections of CS exposure mouse and control mouse. B. (B-1, B-2) The expression of Bcl-2, Bax, and cleaved caspase 3 between lung tissue of CS exposure mouse and control mouse detected by Western blot. *, $P < 0.05$.

Supplementary Figure 3. The immunofluorescence staining of lung tissue of CS exposure mouse and control mouse with TUNEL and endothelial cell marker CD31, 2023

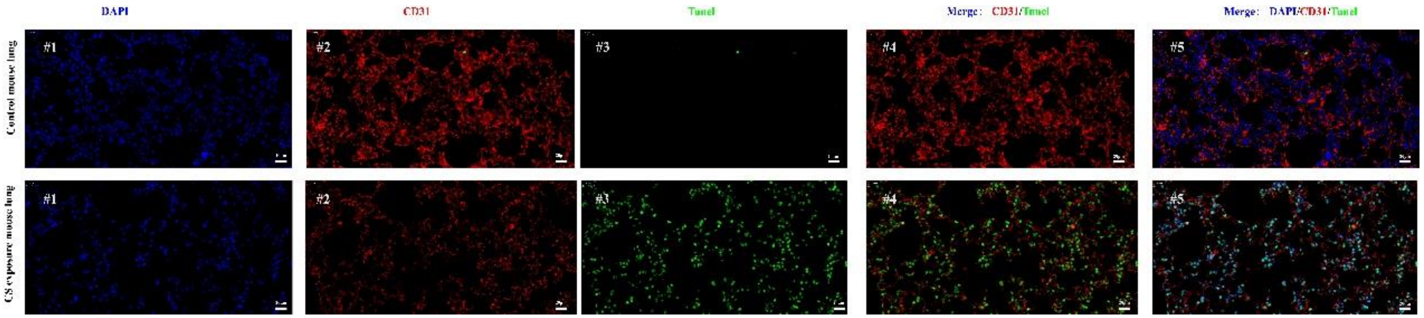

Abbreviations: Immunofluorescence staining of lung tissue samples with TUNEL (green), endothelial cell marker CD31 (red), and DAPI (blue). Scale bar, 20 µm (×400).

Supplementary Figure 4. LRG1 is over-expressed in PMVESs of CS exposure mouse, 2023

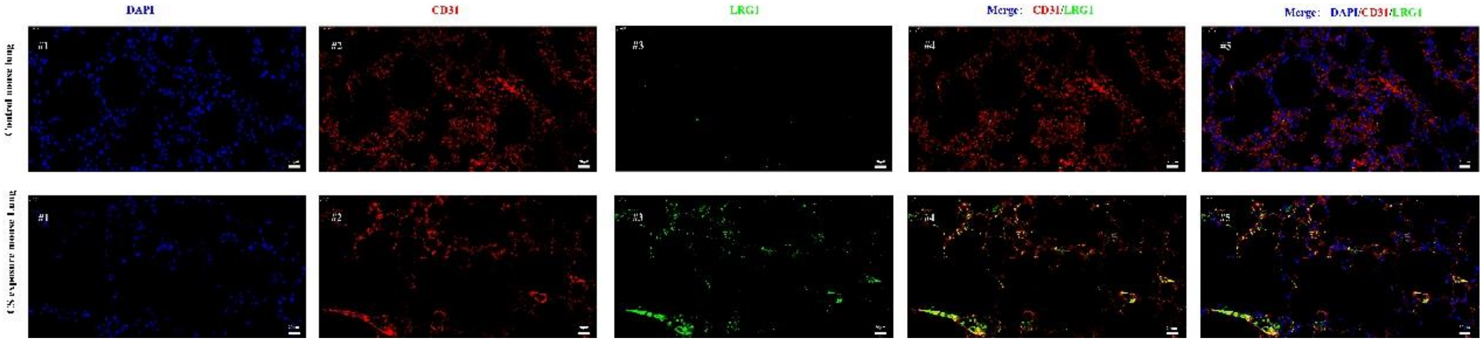

Abbreviations: Representative images of control mouse and CS exposure mouse lung sections stained for LRG1 (green), CD31 (red), and DAPI (blue). Scale bar, 20 μm (×400).

Supplementary Figure 5. The light microscopic images and purity identification of primary mouse PMVECs, 2023

A

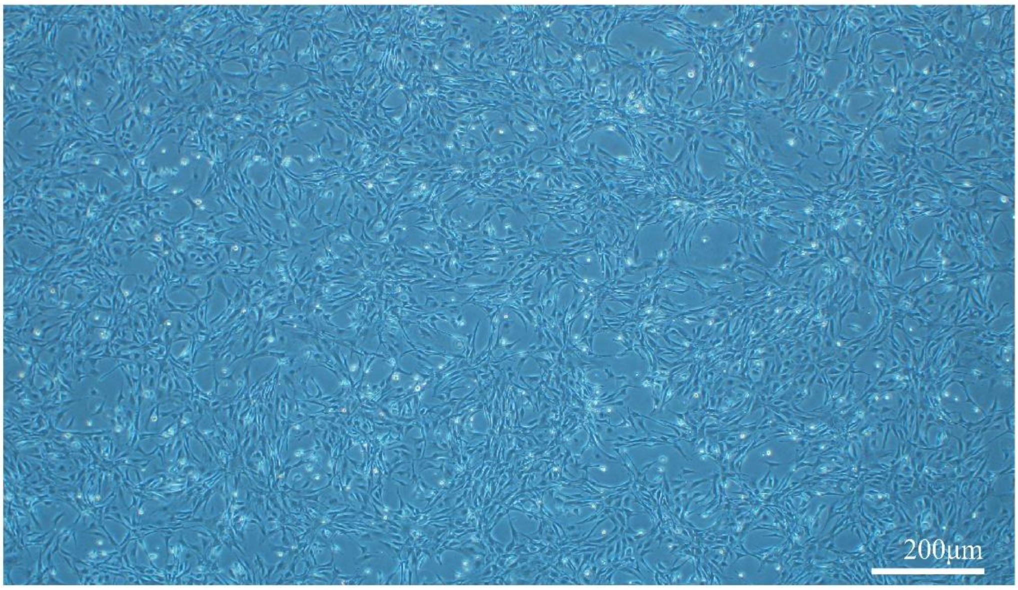

B

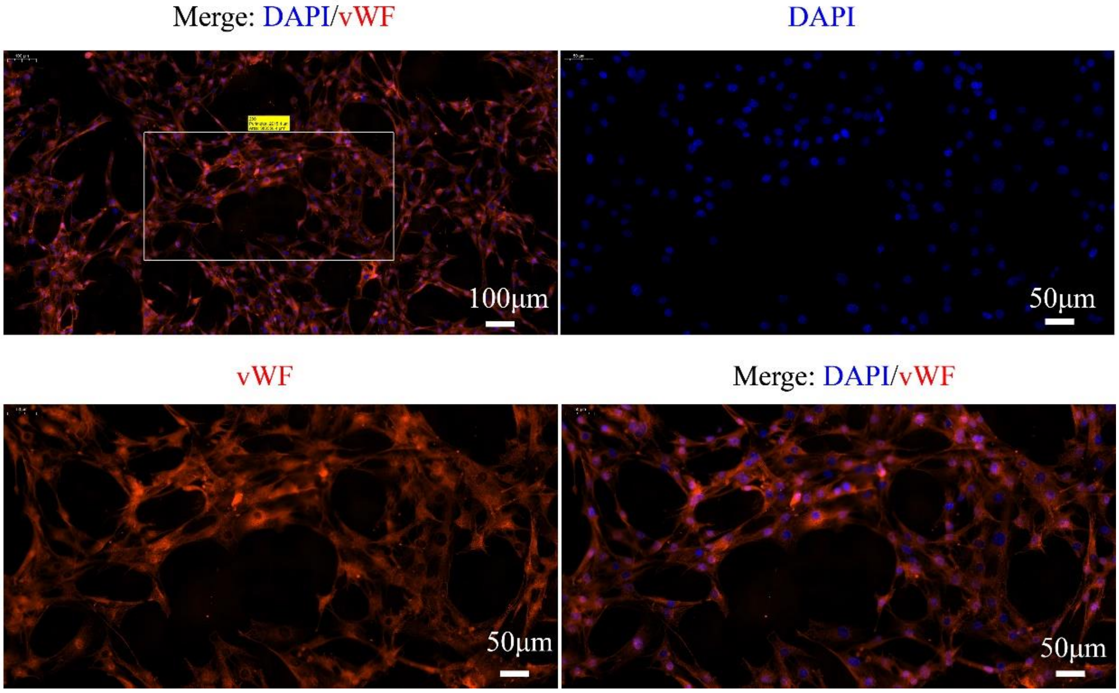

Supplementary Figure 6. The apoptosis is induced by CSE intervention in mouse PMVECs, 2023

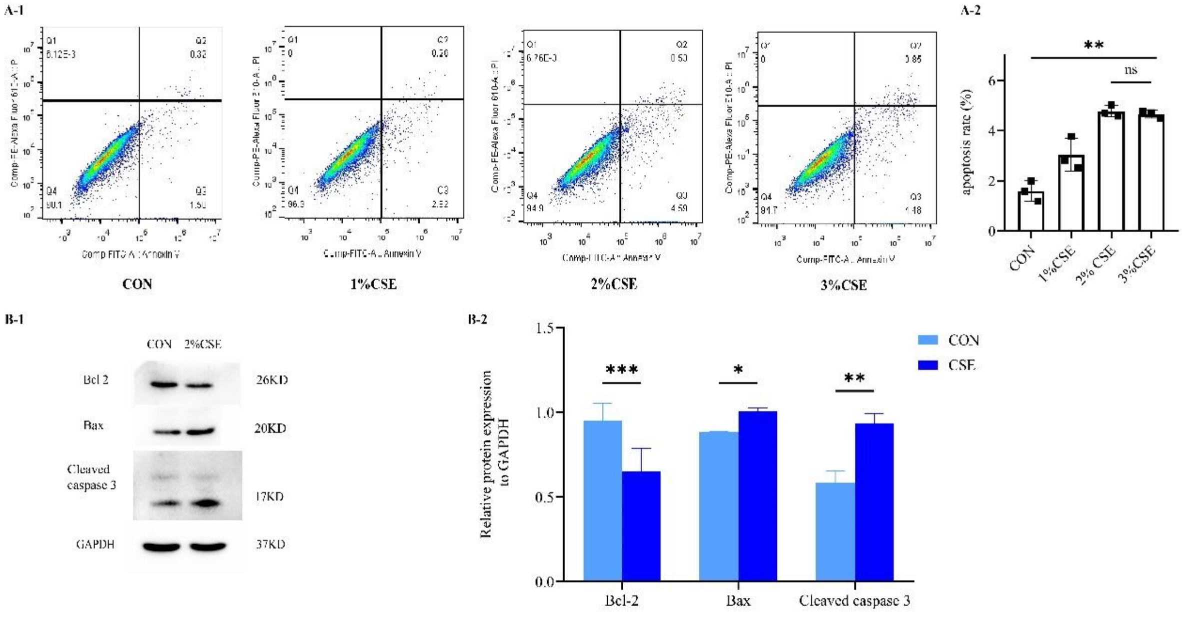

Abbreviations: A. (A-1, A-2) Different concentrations of CSE for 24 hours on cell apoptosis in PMVECs measured by flow cytometry. B. (B-1, B-2) The expression of Bcl-2, Bax, and cleaved caspase 3 between the control group and 2% CSE intervention group in PMVECs detected by Western blot. ns, not significant, *, $P < 0.05$, **, $P < 0.01$, ***, $P < 0.001$.

Supplementary Figure 7. The function of LRG1 in mouse PMVECs measured by flow cytometry, 2023

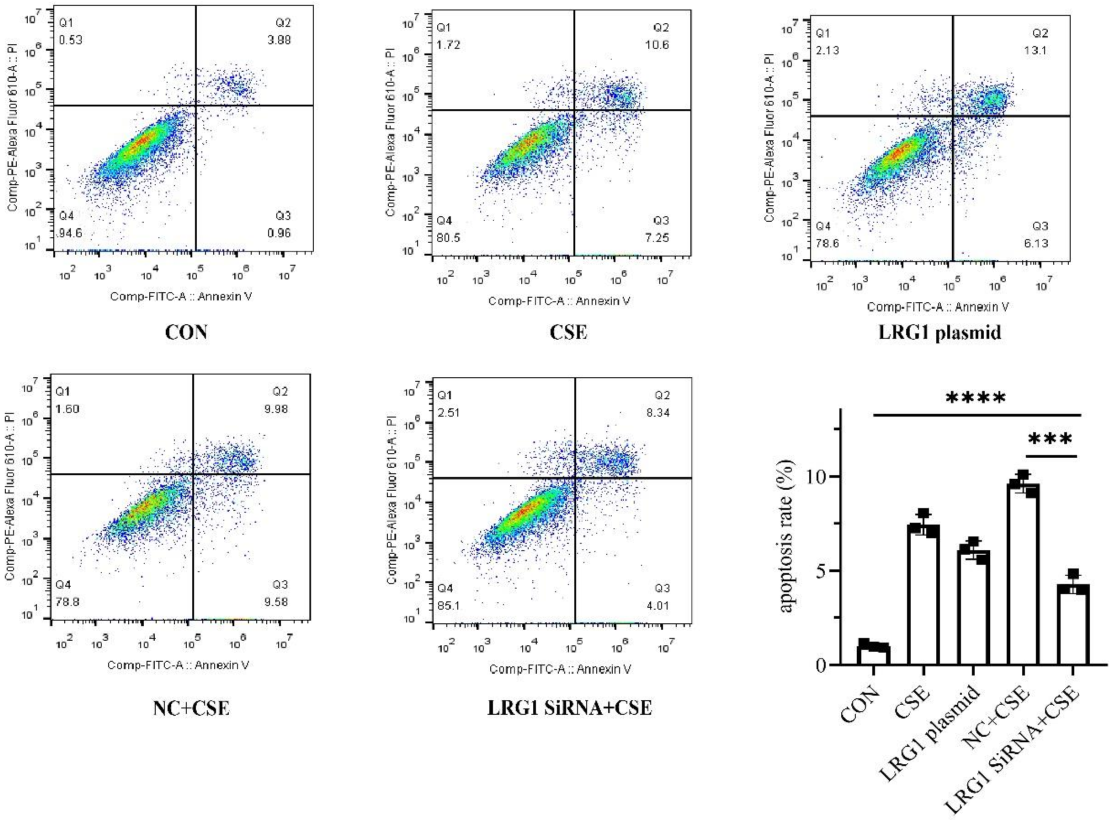

Abbreviations: Apoptosis rate in PMVECs between the control group, CSE group, LRG1 plasmid group, negative control (NC) plus CSE group, and LRG1 SiRNA plus CSE group measured by flow cytometry. ***, $P < 0.001$ (NC+CSE compared with LRG1 SiRNA+CSE); ****, $P < 0.0001$ (control group compared with other four groups, respectively).

Supplementary Figure 8. The knockdown identification of LRG1 in mouse PMVECs, 2023

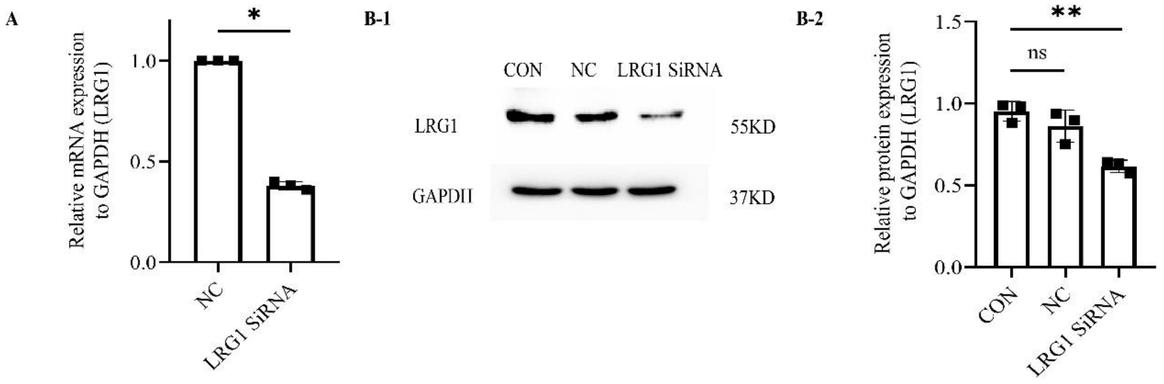

Abbreviations: A-B. Knockdown identification of LRG1 mRNA (A) and protein (B-1, B-2) after transfecting LRG1 SiRNA for 72 hours with mouse PMVECs. ns, not significant, *, P < 0.05, **, P < 0.01.

Supplementary Figure 9. The colocalization of LRG1 and KLK10 in lung tissues extracted from patients and mice, 2023

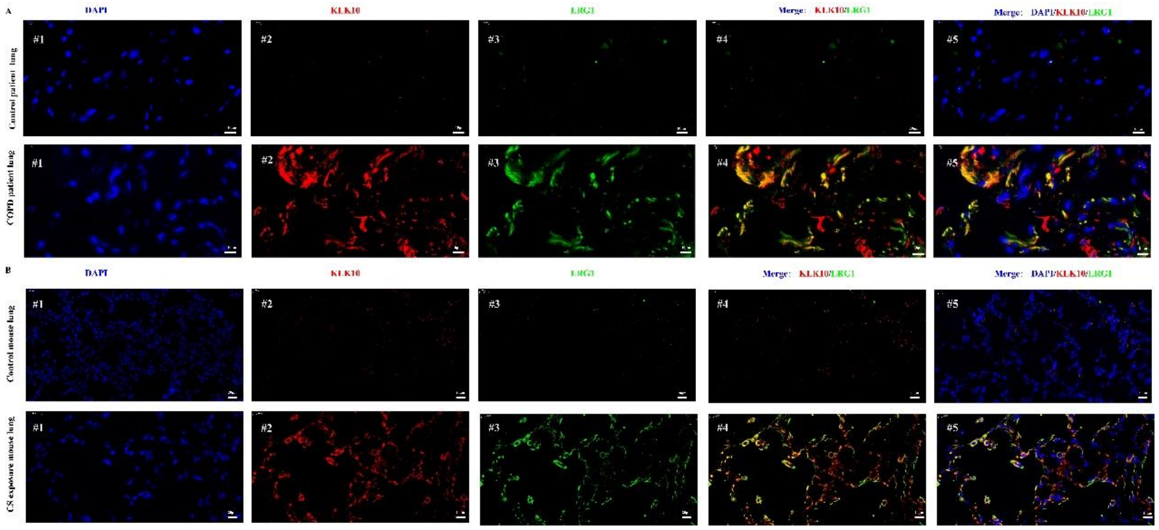

Abbreviations: A. Representative images of control non-COPD and COPD patient's lung sections stained for LRG1 (green), KLK10 (red), and DAPI (blue). B. Representative images of control mouse and CS exposure mouse lung sections stained for LRG1 (green), KLK10 (red), and DAPI (blue). Scale bar, 20 μm (×400). Scale bar, 10 μm (×1000).

Supplementary Figure 10. KLK10 is over-expressed in lung tissue of COPD patients and CS exposure mice, 2023

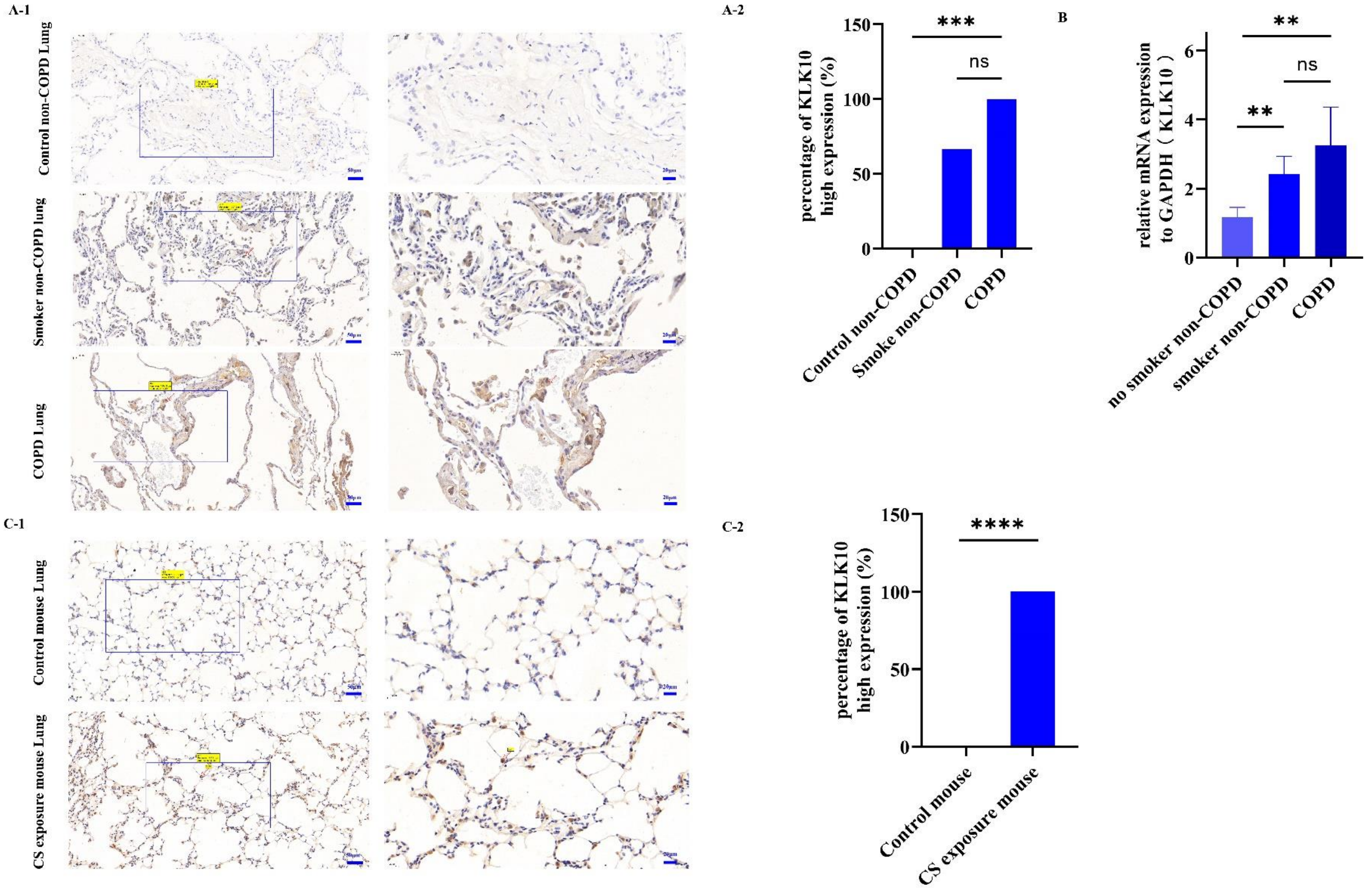

Abbreviations: A. (A-1) The expression of KLK10 between lung tissue of control non-COPD, smoker non-COPD, and COPD detected by immunohistochemistry (n=3 per group). Scale bar, 50 μm (×200); Scale bar, 20 μm (×400). The red arrow points to the brown portion were KLK10. (A-2) Statistical analysis of KLK10 high protein expression between lung tissue of control non-COPD, smoker non-COPD, and COPD detected by immunohistochemistry. B. Quantification of KLK10 mRNA expression between lung tissue of control non-COPD (n=7), smoker non-COPD (n=7), and COPD (n=5) detected by qPCR. C. (C-1) The expression of KLK10 between lung tissue of control mouse and CS exposure mouse detected by immunohistochemistry (n=3 per group). Scale bar, 50 μm (×200); Scale bar, 20 μm (×400). The red arrow points to the brown portion were KLK10. (C-2) Statistical analysis of KLK10 high protein expression between lung tissue of control mouse and CS exposure mouse detected by immunohistochemistry. ns, not significant, *, $P < 0.05$, **, $P < 0.01$, ***, $P < 0.001$, ****, $P < 0.0001$.

Supplementary Figure 11. The colocalization of KLK10 in the pulmonary endothelial population, 2023

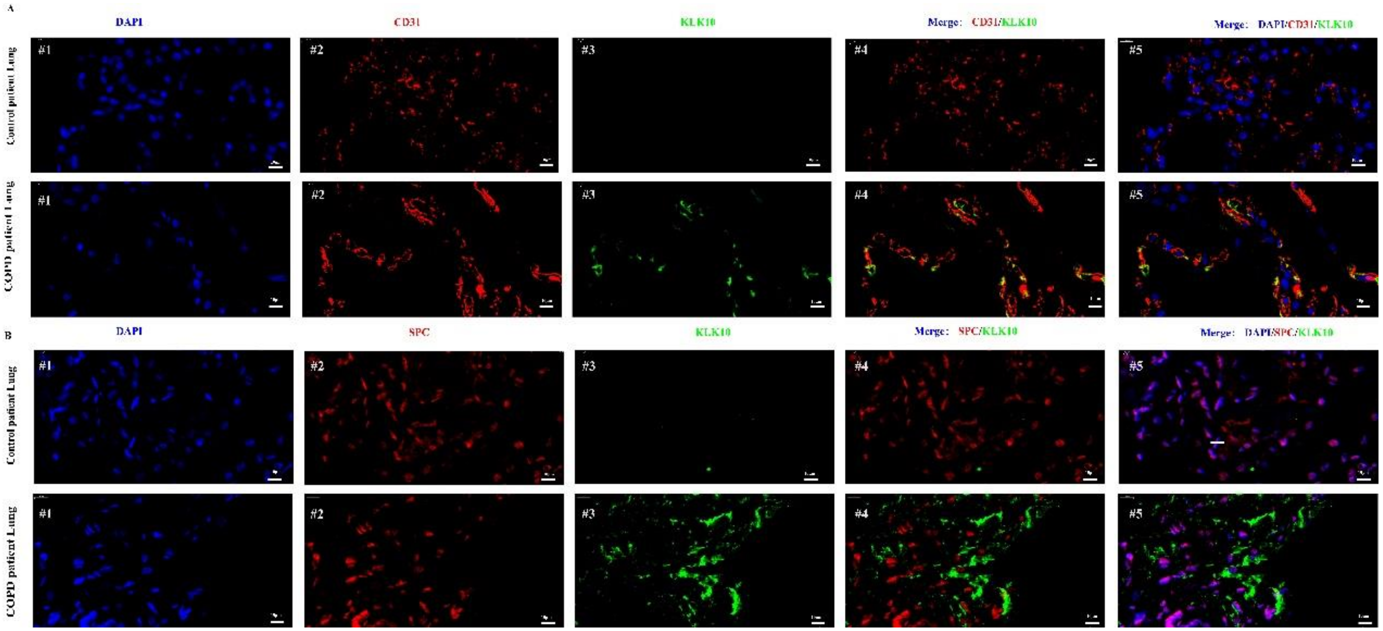

Abbreviations: A. Representative immunofluorescent images of control non-COPD and COPD lung sections stained for KLK10 (green), CD31 (red), and DAPI (blue). B. Representative images of control non-COPD and COPD lung sections stained for KLK10 (green), SPC (red), and DAPI (blue). Scale bar, 10 μm (×1000).

Supplementary Figure 12. The expression of KLK10 in mouse PMVECs treated with CSE and the knockdown identification of KLK10, 2023

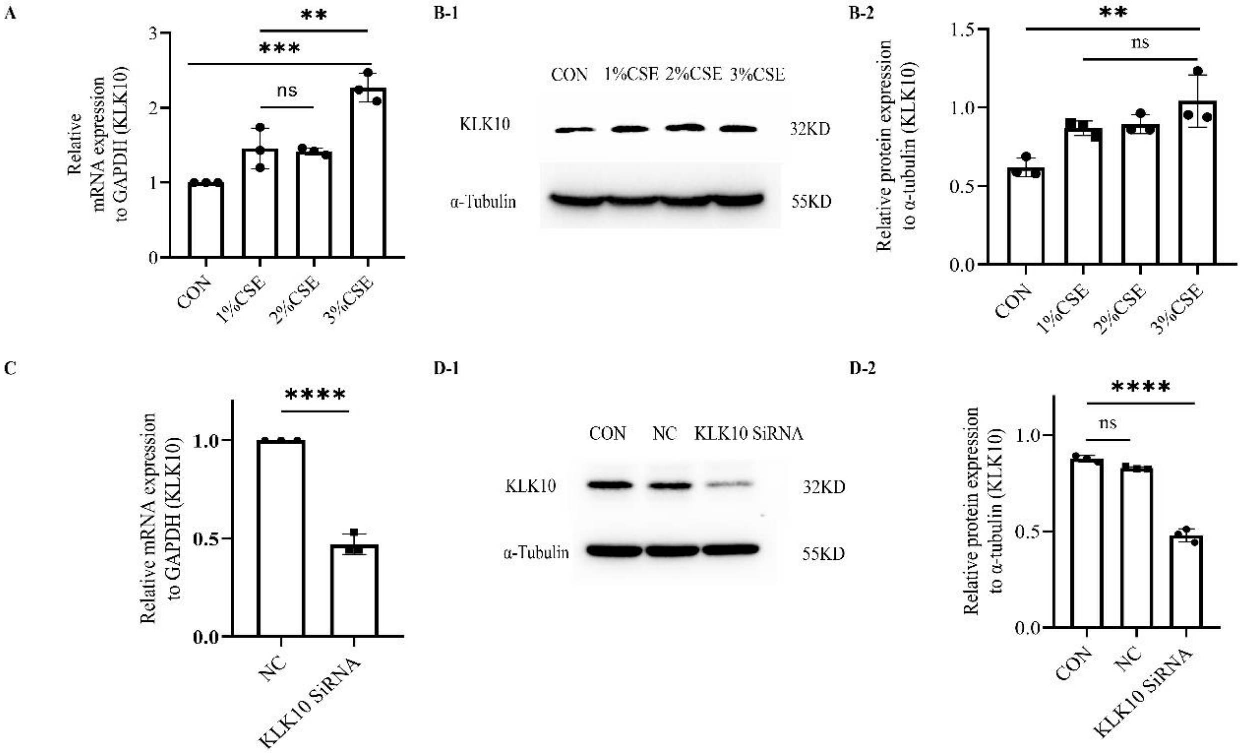

Abbreviations: A-B. The KLK10 mRNA (A) and protein (B-1, B-2) expression in different concentrations of CSE in PMVECs. C-D. Knockdown identification of KLK10 mRNA (C) and protein (D-1, D-2) after transfecting KLK10 SiRNA for 72 hours with mouse PMVECs. ns, not significant, **, $P < 0.01$; ***, $P < 0.001$, ****, $P < 0.0001$.

Supplementary Figure 13. The research mechanism diagram, 2023

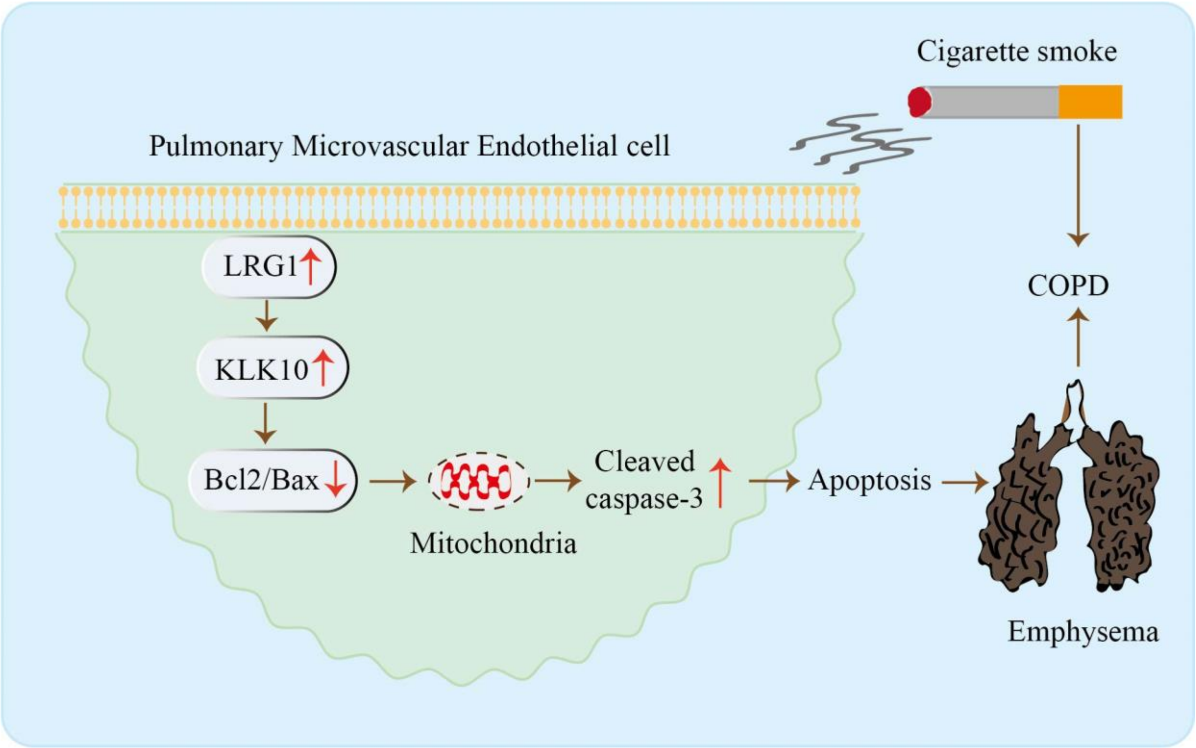

Supplementary Figure 14. The expression of NF-kB in the lung tissue of COPD patient and CS exposure mouse, 2023

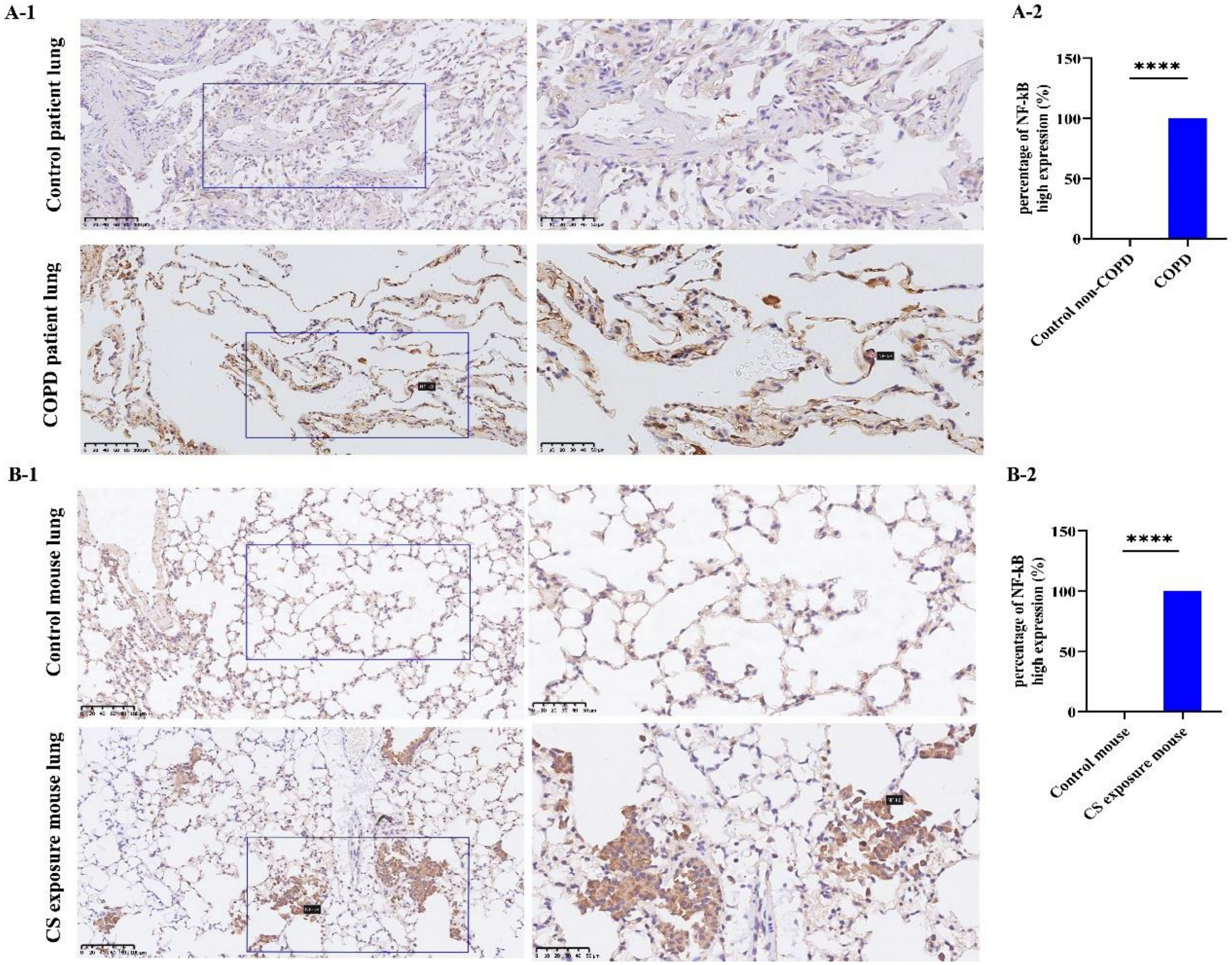

Abbreviations: A. (A-1) The expression of NF-kB between lung tissue of control non-COPD and COPD detected by immunohistochemistry (n=3 per group). Scale bar, 100 μm (right panel, ×200). Scale bar, 50 μm (left panel, ×400). The red arrow points to the brown portion were NF-kB. (A-2) Statistical analysis of KLK10 high protein expression between lung tissue of control non-COPD and COPD detected by immunohistochemistry. B. (B-1) The expression of NF-kB between lung tissue of control mouse and CS exposure mouse detected by immunohistochemistry (n=3 per group). Scale bar, 100 μm (right panel, ×200). Scale bar, 50 μm (left panel, ×400). The red arrow points to the brown portion were NF-kB. (B-2) Statistical analysis of NF-kB high protein expression between lung tissue of control mouse and CS exposure mouse detected by immunohistochemistry. ****, $P < 0.0001$.

Supplementary Figure 15. The colocalization of NF-kB in the pulmonary endothelial population in lung tissues extracted from patients and mice, 2023

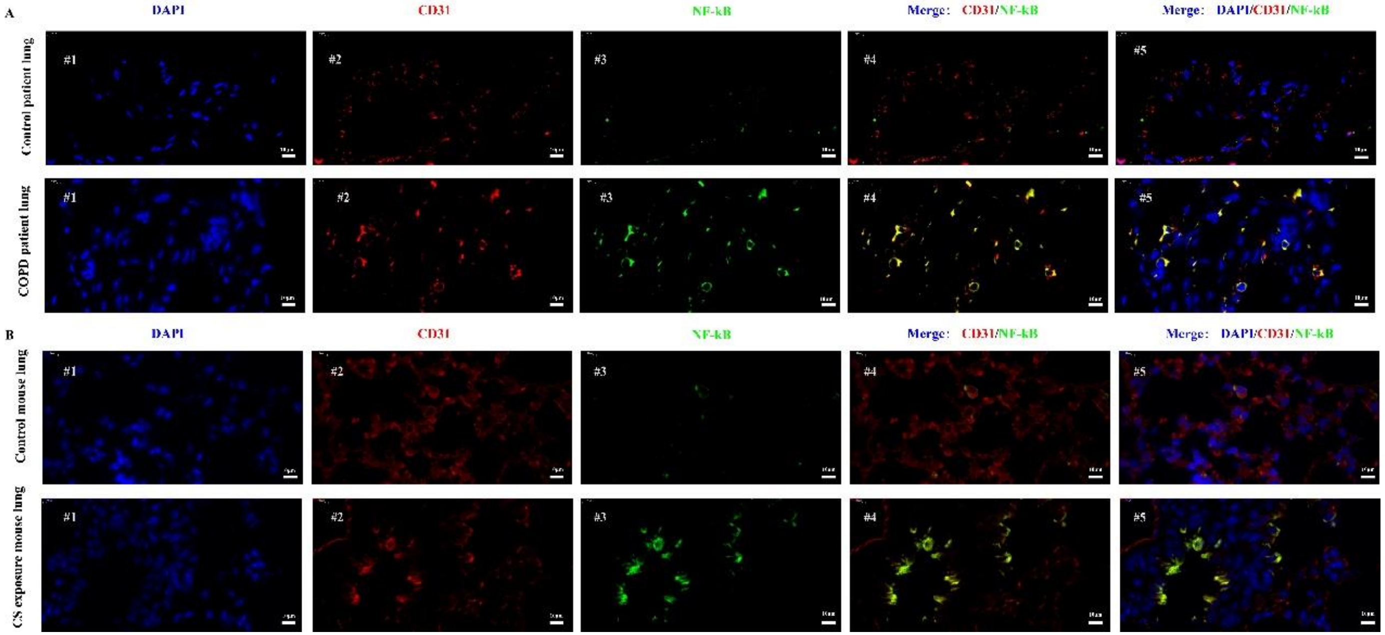

Abbreviations: A. Representative images of control non-COPD and COPD patient's lung sections stained for NF-kB (green), CD31 (red), and DAPI (blue). B. Representative images of control mouse and CS exposure mouse lung sections stained for NF-kB (green), CD31 (red), and DAPI (blue). Scale bar, 10 μm (×1000).

©2024 Cheng W. et al.
